# Discrepancy between Exercise Performance, Body Composition, and Sex Steroid Response after a Six-Week Detraining Period in Professional Soccer Players

**DOI:** 10.1371/journal.pone.0087803

**Published:** 2014-02-19

**Authors:** Nikolaos E. Koundourakis, Nikolaos E. Androulakis, Niki Malliaraki, Christos Tsatsanis, Maria Venihaki, Andrew N. Margioris

**Affiliations:** 1 Department of Clinical Chemistry-Biochemistry, University of Crete, School of Medicine, Heraklion, Greece; 2 Department of Hematology Laboratory, University Hospital, Heraklion, Greece; 3 Departments of Clinical Chemistry-Biochemistry, University of Crete, School of Medicine, and University Hospital, Heraklion, Greece; Tor Vergata University of Rome, Italy

## Abstract

**Purpose:**

The aim of this study was to examine the effects of a six-week off-season detraining period on exercise performance, body composition, and on circulating sex steroid levels in soccer players.

**Methods:**

Fifty-five professional male soccer players, members of two Greek Superleague Teams (Team A, n = 23; Team B, n = 22), participated in the study. The first two weeks of the detraining period the players abstained from any physical activity. The following four weeks, players performed low-intensity (50%–60% of VO_2_max) aerobic running of 20 to 30 minutes duration three times per week. Exercise performance testing, anthropometry, and blood sampling were performed before and after the six-week experimental period.

**Results:**

Our data showed that in both teams A and B the six-week detraining period resulted in significant reductions in maximal oxygen consumption (60,31±2,52 vs 57,67±2,54; p<0.001, and 60,47±4,13 vs 58,30±3,88; p<0.001 respectively), squat-jump (39,70±3,32 vs 37,30±3,08; p<0.001, and 41,05±3,34 vs 38,18±3,03; p<0.001 respectively), and countermovement-jump (41,04±3,99 vs 39,13±3,26; p<0.001 and 42,82±3,60 vs 40,09±2,79; p<0.001 respectively), and significant increases in 10-meters sprint (1,74±0,063 vs 1,79±0,064; p<0.001, and 1,73±0,065 vs 1,78±0,072; p<0.001 respectively), 20-meters sprint (3,02±0,05 vs 3,06±0,06; p<0.001, and 3,01±0,066 vs 3,06±0,063; p<0.001 respectively), body fat percentage (Team A; p<0.001, Team B; p<0.001), and body weight (Team A; p<0.001, Team B; p<0.001). Neither team displayed any significant changes in the resting concentrations of total-testosterone, free-testosterone, dehydroepiandrosterone-sulfate, Δ4-androstenedione, estradiol, luteinizing hormone, follicle-stimulating hormone, and prolactin. Furthermore, sex steroids levels did not correlate with exercise performance parameters.

**Conclusion:**

Our results suggest that the six-week detraining period resulted in a rapid loss of exercise performance adaptations and optimal body composition status, but did not affect sex steroid resting levels. The insignificant changes in sex steroid concentration indicate that these hormones were a non-contributing parameter for the observed negative effects of detraining on exercise performance and body composition.

## Introduction

Soccer periodization typically incorporates a transition – off-season period of reduced stress in order to allow physical and mental recovery after the end of the competition season [1]. This phase of reduction or complete training cessation has been defined as detraining [2]. This detraining period can partial or complete reverse the adaptations of training, resulting in compromised exercise performance [2], [3]. The magnitude of the alterations in performance capacity is dependent on several factors, such as the selected recovery strategy, the duration of the detraining phase, and the initial fitness level of the participants [1].

Evidence from various athletic populations indicate that 3 to 6 weeks of detraining affects negatively aerobic capacity [4], [5], strength [6], [7], neuromuscular performance [7], [8], and body composition [8], [9]. In contrast, some studies on recreational athletes and untrained individuals failed to support these findings in regard to aerobic capacity and muscle strength. It was observed that training cessation or insufficient training stimulus for a period of 2 to 6 weeks did not result in decrements in these two parameters [2], [3], [7], [10]. These discrepancies in the literature were attributed to the different initial training fitness levels of the participants. It has been suggested that the higher the training state of the participants, the greater the rate of decline in both VO_2_max and strength adaptations [4], [7].

This negative effect of detraining on exercise performance adaptations is a result of complex physiological mechanisms. It has been reported that the decline in aerobic capacity is related to reductions in blood volume, stroke volume, cardiac output, ventilator functions, and cardiac dimension due to insufficient training stimulus or training cessation [2], [3]. Similarly, the observed detraining related decrease in muscle strength and strength related performance appears to be a result of decreases in muscle fiber size, mostly due to reduced type II muscle fiber area, mitochondrial ATP production, and enzymatic activities [2], [3].

Few studies have examined the detraining effects on the neuroendocrine system. Even less evidence exists for the relationship between detraining and the male reproductive system. The suggestion that sex hormones are related with physical activity and various physiological systems in the body [11], raise the question of their response to detraining periods. Generally, manipulation of the training regime results in alterations of the neuroendocrine system [7]. The majority of the available evidence shows no significant differences in testosterone, luteinizing hormone, and follicle-stimulating hormone after detraining [7], [12], [13]. However, it has been observed that a short detraining period of a two-week duration increase total testosterone resting levels [14]. It was suggested that the first two weeks of detraining, after stressful training periods, total testosterone levels increase either significantly or insignificantly, indicating increased anabolism and this response is attenuated after the forthcoming 3 weeks of the detraining period [13].

In soccer, little interest has focused on the off-season period. The limited available data suggests that the off-season detraining period results in reductions in aerobic capacity, sprinting ability, increased body weight, and body fat percentage in both professional and semi-professional soccer players [8], [9], [15]. To the best knowledge of the authors, no available data exists in regard to jumping ability and hormonal responses in professional soccer players during this transition period.

Therefore, the **aim of our study** was to examine the effects of a six-week detraining period in professional Greek male soccer players on aerobic capacity, jumping ability, sprint performance, body composition, and sex steroids. More specifically, we tested soccer players for maximal oxygen consumption (VO_2_max), squat jump (SJ), countermovement jump (CMJ), 10 meters (10 m), and 20 meters (20 m) sprint performance. Regarding sex steroids, we evaluated the responses of the most important androgens, total testosterone (TT) and free testosterone (FT), the precursors of testosterone, Δ4-androstenedione (Δ4) and dehydroepiandrosterone sulfate (DHEAS), the metabolic product of their activated form and indicator of the androgen pool [16] 3α-Androstanediol Glucuronide (3a Diol G), and the main estrogens i.e. estradiol (E2), luteinizing hormone (LH), follicle-stimulating hormone FSH, and prolactin (PRL). We hypothesized that all performance parameters and body composition variables will be negatively affected, as a result of the insufficient training stimulus over the six-week detraining period, whereas no hormonal alterations will be revealed.

## Materials and Methods

### Participants

Fifty five professional male Greek football players, members of two Superleague teams (Team A, n = 23; Team B, n = 22) were selected and participated in this study. The mean values for age (years) ± SD and height (cm) ± SD in teams A and B were: age = 25,5±5,3; height = 1,82±0,11 and age = 24,7±4,9; height = 1,81±0,07 respectively. The exclusion criteria were as follow: a) any medical or endocrine disorder that could affect the ability of the players to participate in the study and/or affect endogenous hormonal production; b) suspicion of the use of exogenous agents; c) players that their contract was ending before the end of the conclusion of the study. As a result of the third criterion (exclusion criterion c) seven players were excluded from the study (four from team A and three from team B).

### Ethics Statement

Before testing, verbal explanation was given to each player, concerning the aim of the study and the testing procedures, and written informed consent was obtained. The study was performed in strict accordance with the ethical guidelines of the Helsinki Declaration and was approved by the Ethical Scientific Committee of the University Hospital of Heraklion, Greece.

### Study Protocol

All players were tested in two different occasions. The first experimental testing took place immediately after the end of the competition period in the middle of May (pre). The second experimental testing was performed at the beginning of July (post), prior to the beginning of the preparation period for the forthcoming season. Each experimental testing consisted of two days of consecutive measurements. The first day of each experimental session anthropometric characteristics were measured at 08:30 am. From 09:00 to 10:30 am, venous blood samples were obtained to determine the concentration of the measured hormones. In the afternoon of the same day (17:00 pm) the players were tested for squat jump (SJ), countermovement jump (CMJ), 10 m, and 20 m sprint performance. The second day of each experimental session our participants were tested for the determination of maximal oxygen consumption (VO_2_max). The measurements for the determination of VO_2_max values started at 09:30 am. All hormonal and exercise performance measurements, during the two experimental sessions, were performed at the same time of the day and players were tested in the same order to avoid any circadian variation on the measured variables. Verbal encouragement was given to all participants, ensuring the maximal effort throughout all testing procedures. Before each experimental session, players were informed not to consume any supplement that could promote performance at least 2 days prior to the testing. Moreover, they were instructed to abstain from any physical activity two days prior to each testing in order to avoid any fatigue effects. All players were familiarized with the testing protocol, as they had been previously tested on several occasions during the last soccer season with the same testing procedures. The weekly training plan, during the last 4 months of the competitive season, was similar for both teams, as it had been previously reported in Greek Superleague teams [17]. This study was a part of a larger research project examining the adaptations of many hormonal, physiological, and biochemical parameters during one year period in professional soccer players.

### Nutritional Guidelines

Detailed nutritional guidelines were given to all players for the whole detraining period. In particular, our participants were instructed to consume a high carbohydrate (>60%), low fat (15–25%), and low protein (10–15%) diet [18]. All players were provided with a list of a variety of foods, which included the caloric and nutrient content (carbohydrate, fat, and protein) of each serving portion [18], [19]. Moreover, each player was provided with a table which included the caloric cost of various daily and physical activities [19] (of different intensities). In addition, the resting metabolic rate of each player was assessed [20]. Based on this information, players were asked to compose a diet as per their daily energy requirements (i.e. according to the performed daily activities and exercise training regimes) in order to meet their calculated daily energy expenditure, but at the same time retaining the basic characteristic of their diet i.e. high carbohydrate, low fat, low protein. Furthermore, representative daily meals of 2000, 2500, 3000, and 3500 kcal were provided to each player. Each daily plan included the quantity of each ingredient, its caloric value, and alternative but equal in calories foods. Finally, players were asked to maintain the optimal hydration status, according to set procedures [18], [21]. The aim of these guidelines were as follow: a) to ensure that the carbohydrate content would be adequate for the training activities performed during the detraining period as well as during the two experimental sessions; b) to ensure that the production levels of endogenous sex steroids would not be altered by the nutritional parameters, and; c) to avoid weight gain and increased fat mass due to excessive energy intake and high fat percentage consumption.

### Detraining Period

A six-week detraining period was set in our study. During the first two weeks of this recuperation period, participants were informed to avoid any kind of physical activity. After this two-week period, they were instructed to perform low intensity (50%–60% of VO2max) aerobic running of 20 to 30 minute total duration (30, 20, 2×15, 3×10, 2×10) three times a week, divided by one day of rest.

### Anthropometric Measurements and Body Composition

Height (cm) was measured using a stadiometer (Charder HM210D, Charder Electronics CO, LTD, Taiwan) and weight (kg) was obtained using an electronic weight scale (Seca Alpha 770, Seca Vogel, Hamburg, Germany). Body fat percentage was assessed by skinfold thickness measurement (Lange Skinfold Caliper, Cambridge Scientific Instruments, Cambridge, UK) using the 4 site formula proposed by Durnin and Womersley [22].

### Neuromuscular Performance

The jumping ability of the soccer players (SJ, CMJ) was assessed with a jumping mat (Powertimer, Newtest, Oy, Finland). Three tests were carried out for each jump type and the best result was used for the analysis. During both SJ (cm) and CMJ (cm), the arms were kept in the iliac crest to minimize their contribution. The players performed SJ starting from a standing position, bending the knees to 90°, stopping for three seconds, and then jumping as high as possible. CMJs were performed starting from a standing position, and players were instructed to jump as high as possible, after a fast preparatory downward eccentric movement. Sprint times for 10 m (sec) and 20 m (sec) were measured with infrared photoelectric cells (Powertimer, Newtest, Oy, Finland). For each sprint type two consecutive measurements were performed from a standing position. All sprints were separated by a two-minute rest period for full recovery to avoid any fatigue effects. The best time of the two trials of each distance was used for the analysis. A standardized, low intensity, fifteen-minute warm up was performed prior to each experimental period.

### Maximal Oxygen Consumption (VO_2_max)

Players were tested for the determination of VO_2_max (ml/kg/min) on a motorized treadmill. Warm up consisted of a six-minute run at 8 km/h. Speed was set at 10 km/h and held constant for 3 minutes. Thereafter, speed was increased by 2 km/h every 3 minutes until 16 m/h and then, speed was increased every 2 minutes until voluntary exhaustion. Then, the initial achievement of VO_2_max was considered as the attainment of at least 2 of the following criteria: (a) a plateau in VO_2_ despite increasing speeds, (b) a respiratory exchange ratio above 1.10, or (c) a maximal heart rate within ±5% of age-predicted HR maximum (220-age). Expired gases were analyzed using a breath-by-breath, automated gas-analysis system (VMAX29, Sensormedics, Yorba Linda, CA). Before each test, flow and volume were calibrated using a 3-L capacity syringe (Sensormedics, Yorba Linda, CA). Gas analyzers were calibrated using 2 tanks of oxygen (O2) and carbon dioxide (CO2) of known concentrations (Sensormedics, Yorba Linda, CA).

### Blood Collection and Analysis

On each test day, venous blood samples were obtained after a period of a ten-minute rest in a lying position. Blood samples were collected in tubes, containing a clot activator and serum gel separator and were centrifuged at 3000 rpm for 10 minutes to separate serum. Serum samples were then stored at −70°C till analysis. All samples were tested in duplicate. Total testosterone (ng/ml), LH (IU/L), FSH (mIU/L), E2 (pg/Ml), and PRL (µg/L) concentrations were measured using AIA 21 fully automated immunoassay analyzer (TOSOH-Eurogenetics Tokyo, Japan). The sensitivity of the assays for TT, E2, FSH, LH, and PRL were 7 ng/ml, 25 pg/ml, 1,0 mlU/ml, 0,2 mlU/ml, and 1,05 respectively. The intra and inter coefficient of variation were 3,1–5,2% and 2,48–5,99% for TT, 2,6–6,1% and 3,8–9,1% for E2, 1,5–2,6% and 4,3–5,6% for FSH, 1,8–2,5% and 2,1–2,7% for LH, and 1,8–2,1% and 2,7–2,9% for PRL.

Free testosterone (pg/ml), 3a-Diol G (ng/ml), Δ4-androstenedione (ng/mL), and DHEAS (µg/mL) concentrations were measured using enzyme-linked immunoabsorbent assays (Alpco Diagnostics, Windham, NH). All procedures were carried out according to the instructions of the manufacturer. The sensitivity of the assays for FT, Δ4-androstenedione, DHEAS, and 3a Diol G were 0,17 pg/ml, 0,04 ng/ml, 0,005 µg/ml, and 0,1 ng/ml respectively. The intra and inter coefficient of variation were 4,7–17% and 5,3–12,4% for FT, 4,9–5,8% and 7,7–9,7% for Δ4-androstenedione, 7,5–11,5% and 4,2–15,3% for DHEAS, and 6,0–7,8% and 6,5–10,8% for 3a Diol G. All samples were analyzed in duplicate and in the same assays.

### Statistical Methods

Statistical analysis was performed using software program SPSS 17.0. Standard statistical methods were used for the determination of means and standard deviations (±SD). The changes between the experimental periods in the measured parameters within the groups were analyzed by the paired samples t-test. Pearson's (for normally distributed variables) and Spearman's (for non-normally distributed variables) correlation coefficients were used to assess the linear relationship between quantative variables. The differences between the groups at baseline, in all hormonal, performance, and body composition parameters were analyzed with the General Linear Model (GLM) analysis of variance, aiming to examine whether both teams had similar values in all these variables at the starting point. Statistical power analysis was performed (Stata® 13 software, StataCorp LP, USA) in order to attain 80% power. Analysis was carried out at a confidence level = 95% and confidence interval = 13,6 [7]. Our calculations directed us to a sample size of 45 to detect any differences in changes of the measured variables between the two experimental sessions. The level of significance was set at p<0.05.

## Results

### Differences between the Groups at Baseline

No significant differences were observed between the two experimental groups at the baseline measurement for VO_2_max (F = 0.23, p = 0.88), SJ (F = 1.84, p = 0.18), CMJ (F = 2.44, p = 0.12), 10 m (F = 0.12, p = 0.73), 20 m (F = 0.15, p = 0.69), body weight (F = 0.016, p = 0.89), and body fat percentage (F = 0.003, p = 0.95). Similarly, no significant differences were observed for any of the TT (F = 0.38, p = 0.53), 3a Diol G (F = 0.28, p = 0.59), FT (F = 2.74, p = 0.10), DHEAS (F = 0.09, p = 0.76), Δ4-androstenedione (F = 1.83, p = 0.18), E2 (F = 2.18, p = 0.14), FSH (F = 1.95, p = 0.16), LH (F = 1.28, p = 0.26), and PRL (F = 2.19, p = 0.14) at baseline between the two experimental groups.

### Body Weight and Body Fat Percentage

Changes in body composition variables are presented in table 1. Body weight increased significantly in both teams A (77,60±5,88 vs 79,13±6,16; p<0.001), and B (77,89±8,75 vs 79,49±8,95; p<0.001) at the end of the study compared to baseline. Similarly, a significant increase in body fat percentage was observed in both teams A (9,2±3,33 vs 11,01±4,11; p<0.001) and B (9,43±3,55 vs 10,40±4,08; p<0.001) at the end of the six-week detraining period.

**Table 1:** Mean values ±SD of Body Composition measurements at baseline (Pre) and at the end of the study (Post).

	Team A		Team B	
	Pre	Post	Pre	Post
**Body Weight (kg)**	77,60±5,88	79,13**±6,16	77,89±8,75	79,49±8,95
**Body Fat %**	9,28±3,33	11,01**±4,11	9,34±3,55	10,40±4,08

** Different from Pre (p<0,001).

### Hormones

Sex steroid values at baseline and after the six-week detraining period are presented in table 2. No significant differences were observed for any of the measured hormones in team A: TT (p = 0.14), FT (p = 0.32), Δ4-androstenedione (p = 0.28), DHEAS (p = 0.13), 3a Diol G (p = 0.40), E2 (p = 0.09), FSH (p = 0.11), LH (p = 0.44), and PRL (p = 0.72), and team B: TT (p = 0.73), FT (p = 0.90), Δ4-androstenedione (p = 0.95), DHEAS (p = 0.052), 3a Diol G (p = 0.50), E2 (p = 0.36), FSH (p = 0.88), LH (p = 0.067), and PRL (p = 0.69) at the end of the six-week detraining period compared to their baseline resting values.

**Table 2:** Mean values ±SD of Sex Steroids concentration at baseline (Pre) and at the end of the study (Post).

Hormones	Team A		Team B	
	Pre	Post	Pre	Post
**3a Diol G (ng/ml)**	8,88±3,01	8,31±2,56	8,40±3,08	8,69±2,87
**TT (ng/dl)**	656,97±136,5	604,98±141,9	679,89±109,7	686,81±124,3
**FT (pg/ml)**	12,50±4,86	13,81±6,13	13,11±14,79	13,9±18,30
**Δ4 (ng/mL)**	1,78±0,46	1,86±0,39	2,07±0,93	2,077±1,26
**DHEAS (µg/mL)**	1,99±0,63	2,35±1,07	2,08±1,38	2,89±1,18
**E2 (pg/mL)**	23,69±11,99	19,62±12,82	28,70±16,04	25,38±11,06
**FSH (mIU/L)**	8,29±7,46	7,41±5,56	5,77±4,08	5,71±3,96
**LH (IU/L)**	4,97±1,95	4,69±1,67	4,38±1,92	4,95±1,71
**PRL (µg/L)**	12,80±7,70	12,25±6,71	9,96±4,74	10,24±5,00

### Exercise Performance

**Figure 1:**
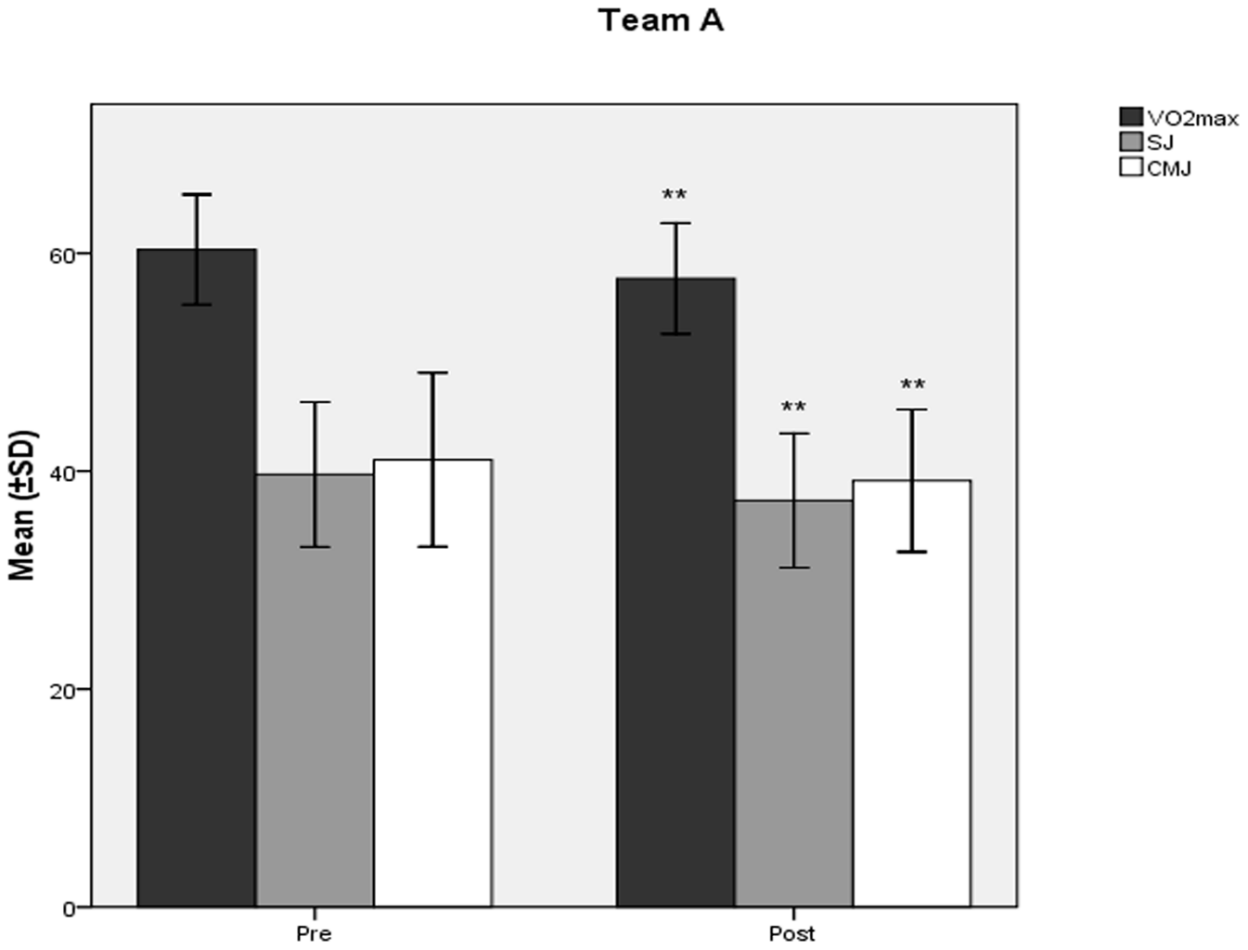
Changes in aerobic and jumping capacity mean values after the detraining period in Team A. Changes (mean values ±SD) in VO_2_max (ml/kg/min), SJ (cm), and CMJ (cm) during the course of the 6 week detraining - off-season soccer period for Team A players. Pre: measurement prior to the beginning of the detraining period; Post: measurement at the end of the detraining period; SJ: squat Jump; CMJ: countermovement Jump. **Different from Pre (p<0.001).

**Figure 2:**
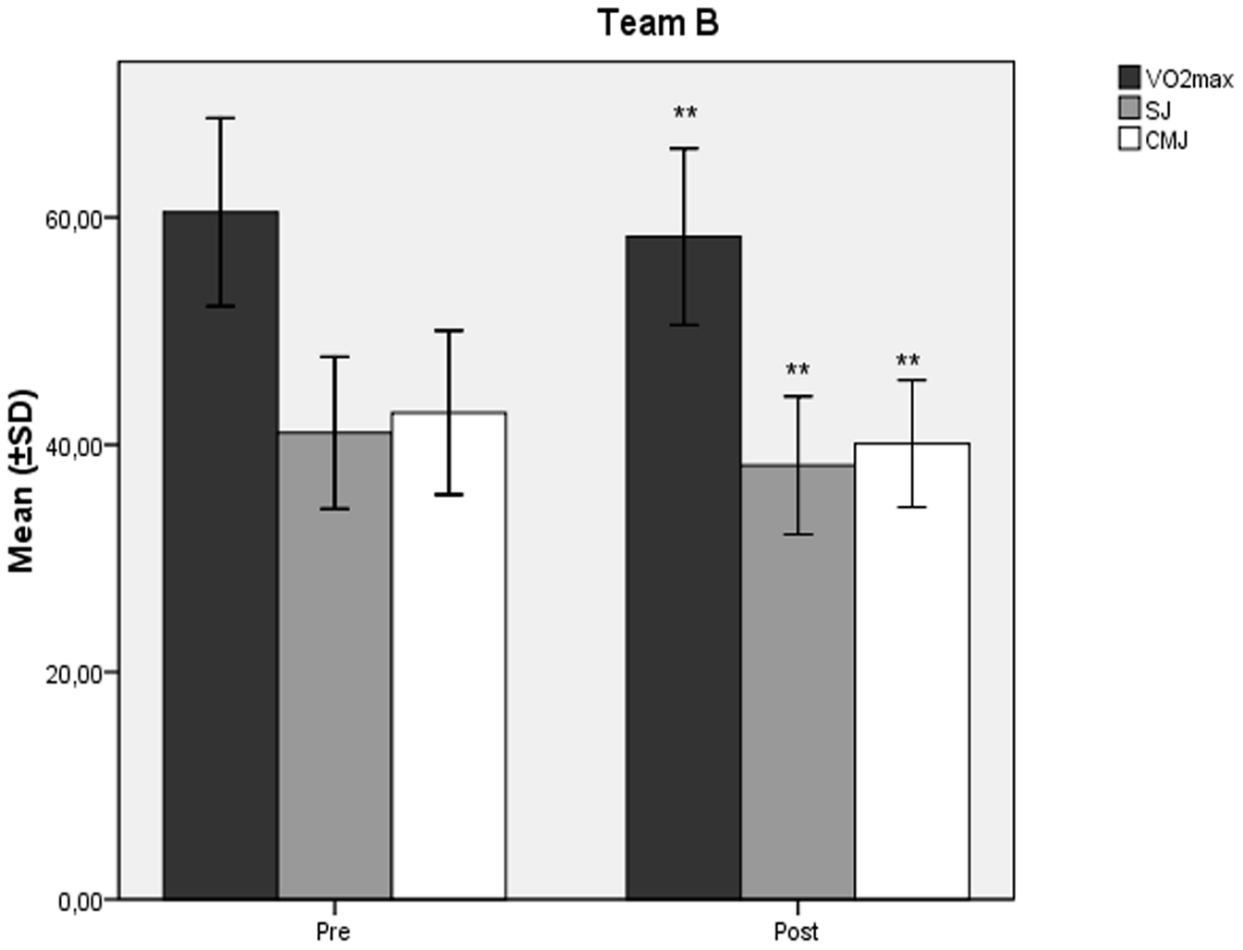
Changes in aerobic and jumping capacity mean values after the detraining period in Team B. Changes (mean values ±SD) in VO_2_max (ml/kg/min), SJ (cm), and CMJ (cm) during the course of the 6 week detraining - off-season soccer period for Team B players. Pre: measurement prior to the beginning of the detraining period; Post: measurement at the end of the detraining period; SJ: squat Jump; CMJ: countermovement Jump. ** Different from Pre (p<0.001).

**Figure 3:**
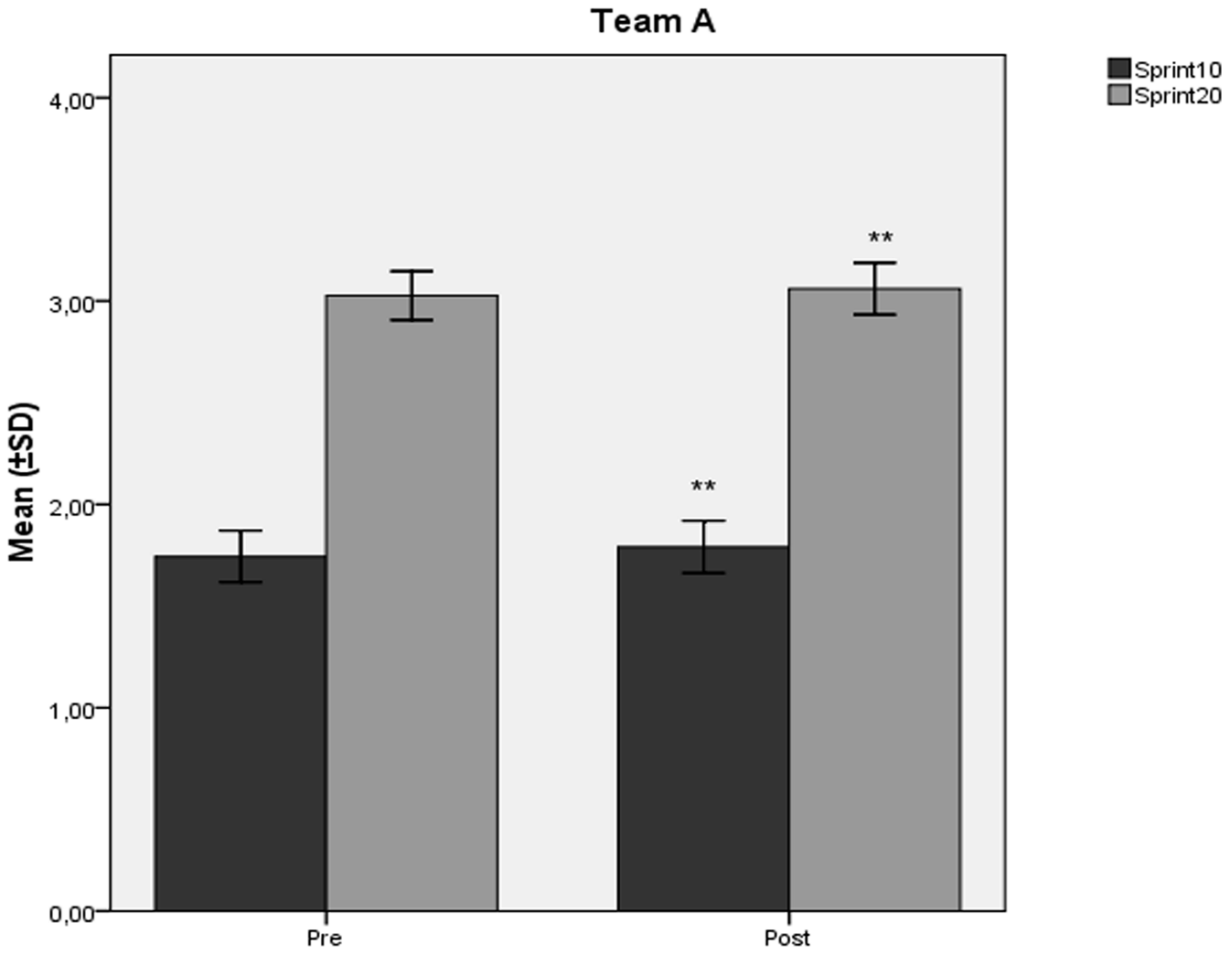
Changes in sprint performance mean values after the detraining period in Team A. Changes (mean values ±SD) in 10 meters (Sprint10) and 20 meters (Sprint20) sprint performance (sec) during the course of the 6 week detraining - off-season soccer period for Team A players. Pre: measurement prior to the beginning of the detraining period; Post: measurement at the end of the detraining period. ** Different from Pre (p<0.001).

**Figure 4:**
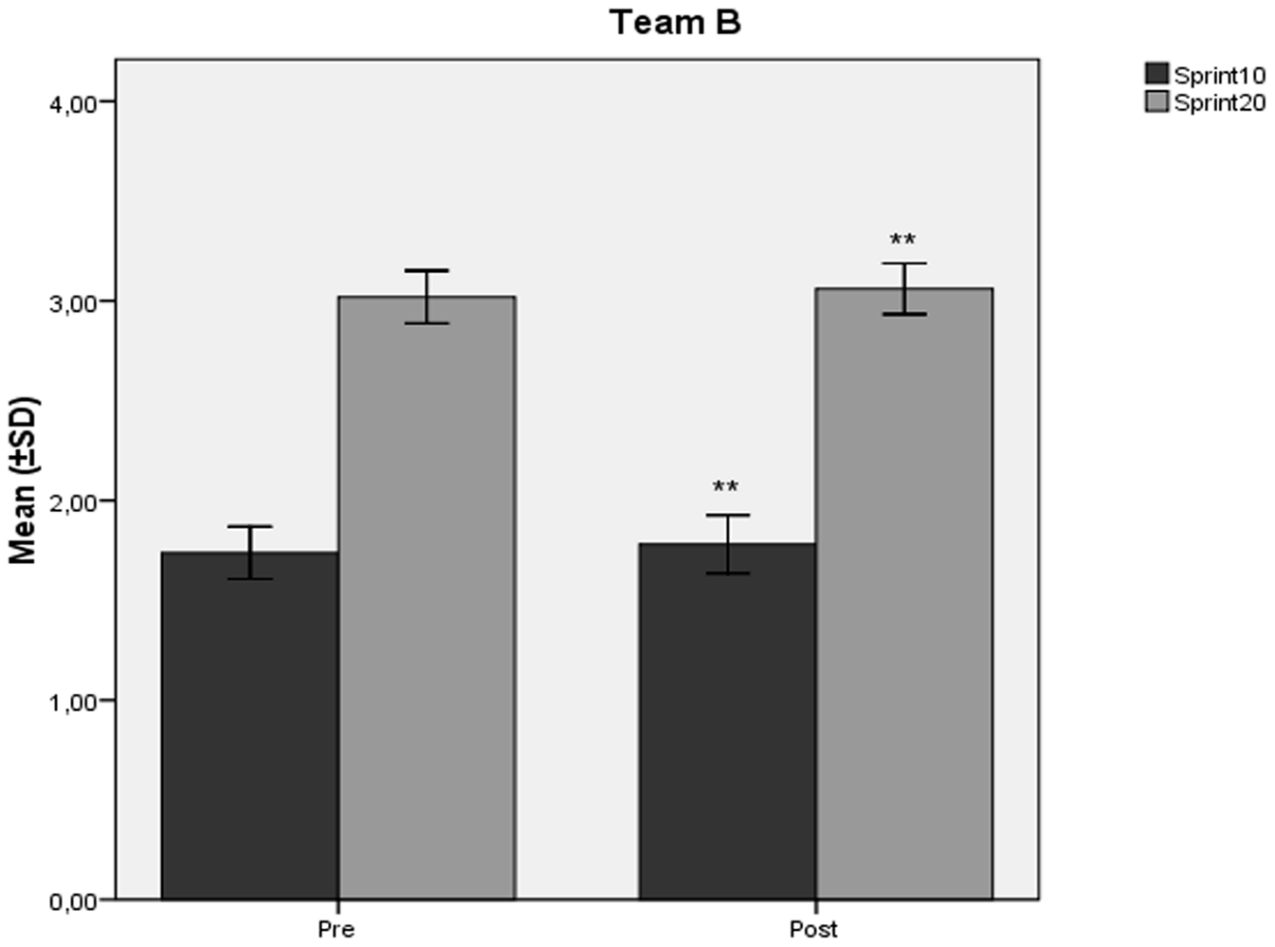
Changes in sprint performance mean values after the detraining period in Team B. Changes (mean values ±SD) in 10 meters (Sprint10) and 20 meters (Sprint20) sprint performance (sec) during the course of the 6 week detraining - off-season soccer period for Team B players. Pre: measurement prior to the beginning of the detraining period; Post: measurement at the end of the detraining period. ** Different from Pre (p<0.001).

The mean values ± SD of VO_2_max (ml/kg/min) decreased significantly at the end of the study compared to baseline (figure 1, figure 2) in team A (60,31±2,52 vs 57,67±2,54; p<0.001) and team B (60,47±4,13 vs 58,30±3,88; p<0.001). Similarly, in teams A and B, there was observed a significant decline (figure 1, figure 2) in SJ (39,70±3,32 vs 37,30±3,08; p<0.001, and 41,05±3,34 vs 38,18±3,03; p<0.001 respectively), and CMJ (41,04±3,99 vs 39,13±3,26; p<0.001, and 42,82±3,60 vs 40,09±2,79; p<0.001 respectively) values (cm). Both teams showed significant increases in 10 m (1,74±0,063 vs 1,79±0,064; p<0.001, and 1,73±0,065 vs 1,78±0,072; p<0.001 respectively), and 20 m (3,02±0,05 vs 3,06±0,06; p<0.001, and 3,01±0,066 vs 3,06±0,063; p<0.001 respectively) sprint times (sec) at the end of the study compared to baseline (figure 3, figure 4).

### Correlations between sex steroids and performance parameters

Correlations between sex steroids and performance parameters, during the beginning and the end of the 6-week detraining period, are presented in table 3 (team A) and table 4 (team B). Analysis of our results did not reveal any significant differences (p>0.05) between 3a Diol G, TT, FT, Δ4-androstenedione, DHEAS, E2, FSH and PRL in neither team A nor B, during both experimental sessions. Significant correlations were observed between LH and VO_2_max at both the first (p = 0.013) and the second (p = 0.010) experimental sessions. Significant correlations were evident in team B, regarding LH levels with both 10 m, and 20 m values at the first (p = 0.022 and p = 0.004 respectively) and the second (p = 0.028 and p = 0.006 respectively) experimental sessions.

**Table 3:** Correlations (p-values) between circulating sex steroids and exercise performance parameters in team A.

Team A	VO_2_max (ml/kg/min)	SJ (cm)	CMJ (cm)	10 m (sec)	20 m (sec)
	Pre	Post	Pre	Post	Pre	Post	Pre	Post	Pre	Post
**3a Diol G (ng/ml)**	0.394	0.368	0.873	0.355	0.833	0.601	0.177	0.203	0.510	0.177
**TT (ng/dl)**	0.513	0.794	0.682	0.151	0.660	0.122	0.851	0.717	0.564	0.800
**FT (pg/ml)**	0.788	0.487	0.741	0.833	0.927	0.451	0.932	0.613	0.540	0.714
**Δ4 (ng/mL)**	0.381	0.699	0.505	0.571	0.312	0.605	0.79	0.362	0.305	0.791
**DHEAS (µg/mL)**	0.714	0.374	0.611	0.222	0.820	0.202	0.799	0.809	0.666	0.275
**E2 (pg/mL)**	0.782	0.615	0.405	0.727	0.465	0.975	0.072	0.321	0.308	0.580
**FSH (mIU/L)**	0.240	0.090	0.866	0.843	0.531	0.723	0.417	0.324	0.711	0.422
**LH (IU/L)**	0.013*	0.010*	0.687	0.543	0.702	0.225	0.767	0.161	0.621	0.064
**PRL (µg/L)**	0.330	0.276	0.634	0.591	0.528	0.417	0.680	0.185	0.660	0.247

* siginifcant diference at the level of significance p<0.05.

**Table 4:** Correlations (p-values) between circulating sex steroids and exercise performance parameters in team B.

Team B	VO_2_max (ml/kg/min)	SJ (cm)	CMJ (cm)	10 m (sec)	20 m (sec)
	Pre	Post	Pre	Post	Pre	Post	Pre	Post	Pre	Post
**3a Diol G (ng/ml)**	0.719	0.075	0.521	0.268	0.196	0.400	0.163	0.327	0.188	0.699
**TT (ng/dl)**	0.911	0.136	0.34	0.718	0.692	0.480	0.728	0.848	0.216	0.863
**FT (pg/ml)**	0.304	0.926	0.65	0.976	0.383	0.879	0.760	0.366	0.602	0.948
**Δ4 (ng/mL)**	0.052	0.052	0.212	0.930	0.765	0.625	0.769	0.464	0.468	0.592
**DHEAS (µg/mL)**	0.480	0.109	0.263	0.143	0.659	0.678	0.960	0.985	0.952	0.967
**E2 (pg/mL)**	0.481	0.564	0.811	0.651	0.645	0.929	0.436	0.496	0.406	0.518
**FSH (mIU/L)**	0.555	0.907	0.312	0.713	0.995	0.165	0.252	0.077	0.228	0.072
**LH (IU/L)**	0.613	0.352	0.266	0.177	0.082	0.051	0.022*	0.028*	0.004**	0.001**
**PRL (µg/L)**	0.290	0.561	0.204	0.546	0.238	0.804	0.688	0.95	0.629	0.710

* siginifcant diference at the level of significance p<0.05,

** siginifcant diference at the level of significance p<0.01.

## Discussion

Our findings support our hypothesis. After the six-week detraining period, we observed significant reductions in aerobic and jumping capacity values, and significant increases in 10 m and 20 m sprint times, body weight, and body fat percentage. These changes occurred with the absence of any significant changes in the circulating sex steroids basal levels. These observations indicate that the employed detraining period, in our study, resulted in a rapid loss of aerobic and neuromuscular performance adaptations in professional soccer players.

### Maximal Oxygen Consumption (VO_2_max)

As expected, VO_2_max was significantly reduced after the six-week detraining period in both teams A and B. In agreement are the findings by Reilly and Williams [23]. The authors reported that an eight-week detraining period in soccer resulted in decreased aerobic capacity, as indicated by the reduced VO_2_max values. Similarly, a study from another laboratory observed that the off-season soccer period resulted in reduced VO_2_max performance in semi-professional soccer players [15]. In agreement are the findings from other athletic populations. Maximal oxygen consumption has been shown to decline in a variety of sports, even with short term detraining (less than 4 weeks), up to 14% [4], [5], [10], [24]. Furthermore, VO_2_max has been found to decrease in highly trained athletes up to 20% after 4 weeks of detraining [5]. However, some studies showed that short term training cessation or drastically reduction in volume and training frequency, in a period of 4 to 5 weeks, did not affect VO_2_max [1], [25], [26]. These discrepancies could be related with the initial training status of the participants and the training regime employed during the detraining period. Coyle and associates [4] suggested that the higher the training state in VO_2_max, the greater the decline in aerobic capacity after detraining periods. Since our participants were professional soccer players with their VO_2_max mean pre-values (60,31 ml/kg/min for team A, 60,47 ml/kg/min for team B) within the suggested range (55–70 ml/kg/min) for high level soccer players [27], this could be the case in our study. Furthermore, it has been suggested that in order to retain aerobic training adaptations during detraining periods, a high intensity aerobic training stimulus (>80% VO_2_max), even once a week, should be performed [26], [28]. This is further supported by the observations of a recent study in top level Kayakers [1]. The authors reported that a period of 5 weeks under reduced training stimulus, which consisted of a moderate (80% VO_2_max), and not a high intensity aerobic regime, did not manage to retain VO_2_max performance. Therefore, according to the aforementioned evidence, the employed low intensity (50%–60% of VO_2_max) aerobic regime that was performed the last 4 weeks of the detraining period in our study could not provide sufficient stimulus to maintain aerobic capacity.

### Jumping Ability

We observed a significant reduction in both CMJ and SJ after the six-week detraining period in teams A and B. These findings are in agreement with the observations of a recent study in semi-professional soccer players. The authors reported that the off-season detraining period can result in a reduction in jumping ability [15]. However, inconsistent findings have been reported by some studies that examined the detraining effects on strength trained individuals [7], [13], [14]. Indeed, Izquierdo and associates [7] observed that 4 weeks of complete training cessation or tapering in strength trained athletes decreased CMJ performance, whereas other studies on the same athletic population showed that a period of two [14] or six [13] weeks of training cessation did not affect vertical jump performance. These discrepancies were attributed to the different strength training status of the participants in these studies, and only the athletes with the higher strength levels [7] experienced decreases in jumping ability. In our study, the effects were rather related to the employed training regime during the off-season period than the strength status of the participants, since soccer players have been generally reported to have lower muscle strength levels compared to strength trained athletes [27]. Our players followed a low intensity aerobic regime in both teams after the 2 first weeks of training cessation. Previous evidence has demonstrated that this kind of activity does not promote jumping ability but on the contrary, it can negatively affect this kind of performance [29]. A further possible explanation for our observations comes from the suggestion that detraining can result in decreased strength after a 3–6 week period, due to a negative effect on type II muscle fibers [6], [7], [12]. Early research has documented that fast twitch muscle fibers are strongly correlated with maximal strength, which is linearly related with jumping ability and explosive actions such as SJ and CMJ [30]. According to these findings, the observed reductions in jumping performance, in our study, could be related to a possible negative effect of the detraining period on fast twitch muscle fibers.

### Sprint Performance

The observed reductions in 10 m and 20 m sprint performance in both teams are in agreement with the only available studies, to our knowledge, that examined the effect of the soccer off-season period on sprinting capacity [8], [31], [32]. The authors observed that detraining resulted in significant reductions in 10 m, 20 m, and 50 m sprint performance. It was suggested that these findings could be related with reductions in the cross-sectional area of type II muscle fibers, negatively altered anaerobic enzymatic activity, and a decline in mitochondrial ATP production [2], [3], [31]. Since sprint performance is related with muscle morphology, enzymatic activity, and ATP production [33], any possible negative changes during the six-week detraining period in any of the aforementioned parameters could be accounted for the observed reductions in sprinting capacity. Furthermore, it is well established that sprint performance depends on the ability to generate power [34] and that power is a product of strength [35], demonstrating a linear relationship between strength levels and sprinting ability. Since both SJ and CMJ are considered to be the most accurate field tests for the determination of the explosive strength level of the lower limps [36], the observed reduction in their levels, at the end of our detraining period, could further justify the decline in sprint performance.

### Body Composition

At the end of the 6-week detraining period, significant increases in body weight and body fat percentage were evident in both teams. In accordance are the findings of other laboratories [8], [9]. It was observed that the off-season soccer period resulted in increased body weight and body fat percentage in the players. However, in these studies, no off-season training sessions were undertaken. On the contrary, our players performed low intensity aerobic trainings during the last 4 weeks of the detraining period. Confirmation to our findings comes from a recent study in competitive swimmers [37]. The authors reported that 35–42 days of detraining, involving light-moderate physical exercise, after a competitive swim season, resulted in significant increases in body weight and body fat percentage. The increased body fat percentage and body weight, in our study, could be attributed to the reduced training stress during the detraining period. The reduced training stimulus could have resulted in a lowering of the metabolic rate per unit of tissue mass, and effectively decreased resting metabolic rate, which could have a negative impact on body composition [8], [37], although these finding are not universal [38]. Furthermore, it has been observed that during detraining periods there is an increase in lipoprotein lipase (LPL) activity, which facilitates free fatty acid deposition on adipose tissue [39]. These suggestions are supported by the unaltered circulating sex steroids levels in our study. Since changes of internal androgen and estrogen levels are affecting body composition [40], resting metabolic rate [41], [42], and lipase activity [43] the observed changes in body composition should be attributed to other causes and not to changes of the sex steroid milieu. Taken together, our data suggest that the insufficient training stimulus during our detraining period could have resulted in a short-term energy surplus, leading to weight gain, characterized by an increase in fat mass, most probably, due to an increased free fatty acid deposition on adipose tissue secondary to an enhanced LPL activity [37], [39].

### Sex Steroids

In the present study, the detraining period did not significantly affect the circulating levels of the measured sex hormones in our experimental teams. To the best of our knowledge, this is the first study examining the hormonal responses during the off-season period in professional soccer players.

### Total Testosterone, Free Testosterone, and 3a Diol G

Our results showed that neither TT nor FT and the active androgen metabolite 3a Diol G were affected by the six-week detraining period. Notably, this is the first study examining the behavior of 3a Diol G in professional athletes, which has been reported to be an excellent marker of activated androgens [16]. In regard to TT and FT, our observations are in accordance with the findings after a four-week detraining period in strength trained individuals [7]. The authors failed to find any significant changes in TT and FT after both training cessation and tapering. Similarly, Kraemer and associates [13] showed that 6 weeks of detraining in recreationally trained men did not affect TT concentration. On the contrary, two weeks of inactivity on strength trained individuals resulted in significantly increased TT levels [14]. The authors suggested that this could indicate an enhanced stimulus for tissue remodeling. We were unable to confirm this finding since, in our study, no measurement was performed at the first two weeks of the off-season period. However, Kraemer and associates [13], based on their observation of an insignificant increase in TT levels after the first 2 weeks in their study, gave a possible explanation of this finding. The authors suggested that the first 2–3 weeks of detraining, after stressful training periods, could possibly result in increased anabolic hormones concentration, and that this increase was attenuated after the following 3–4 weeks of detraining. The absence of an alteration in 3a Diol G levels, in our study, clearly indicates that no anabolic trend was evident at the end of the six-week detraining period, since this hormone is the metabolic product of androgens and an indicator of activated androgens [16]. In other words, its unchanged resting values at the end of the off-season period clearly states that there was not any enhanced muscle tissue remodeling or any other extra-splanchnic utilization of the circulating androgens.

### Gonadotropins

In regard to LH and FSH, our findings are in accordance with the observations of a study on resistance trained males [12]. The authors reported that 12 weeks of detraining did not affect these two gonadotropins resting levels. Similarly, Hal and associates [44] reported that LH and FSH were not altered by a short detraining period in endurance athletes. Therefore, according to the aforementioned published reports and our own findings, we could propose that detraining does not affect the hypothalamus or the pituitary gland regarding these two hormones.

### Other Hormones

In the published bibliography no evidence exists for E2, Δ4-androstenedione, and PRL and their responses after a detraining period. In regard to DHEAS, only one recent study examined its behavior after 2 months of training cessation in highly trained badminton players [45]. The authors observed a significant reduction in its resting levels at the end of the study. However, we were unable to confirm these observations. Indeed, we have found that the detraining period did not decrease DHEAS concentration, on the contrary, it showed a tendency to increase albeit in a non-significant manner in both experimental teams (Table 2). This discrepancy could be related to the training status of the participants and the training regime used, which have been both reported to affect the hormonal responses to exercise [46].

A possible explanation for the absence of an alteration, not only in regard to DHEAS but also for Δ4-androstenedione, E2, and PRL is their reported correlation with TT levels. It has been suggested that DHEAS and Δ4-androstenedione were linearly correlated with TT levels although these findings are not universal [47], [48]. Similarly, studies on elderly individuals showed the E2 concentration was correlated with its major precursor (TT) levels, in a linear manner [49]. Therefore, the insignificant changes in TT levels at the end of the study could explain the lack of an alteration in E2, Δ4-androstenedione, and DHEAS levels. In regard to PRL, it has been previously reported to have a strong reverse correlation with TT levels [50] in athletic population, indicating that the unaffected TT basal levels resulted in its unaltered resting values.

It should be stressed here that our overall findings, regarding the alterations of the examined performance parameters and the unaffected sex steroids levels, do not appear to be mediated by changes of nutritional intake since the latter was kept unchanged throughout the study. Indeed, during the six-week detraining period we paid great attention to exclude nutritional changes (including that of hydration) by retaining a constant level of a daily diet composed of high carbohydrates, low fat and, low protein consumption, and a religious matching of their energy intake to the calculated level of daily energy expenditure. It should be mentioned that both the carbohydrate content of the diet [51], [52] and energy restriction [53] play regulatory role in the levels of sex steroids. In addition, reduced carbohydrate content of a diet, insufficient energy intake, and dehydration negatively influence the ability of the players to perform efficiently during aerobic and high intensity intermittent exercise activities [21], [54]. Therefore, because of the aforementioned adjustments of each player's dietary intake during the detraining period, we had no measurable changes of the hormonal milieu of their body, and the observed changes in exercise performance capacity should be attributed to the reduction of training.

Finally, no correlations were evident between performance parameters and circulating androgen (TT, FT, 3a Diol G, Δ4-androstenedione, and DHEAS) during the detraining period. Similarly, no correlations were evident between performance and E2, FSH, and PRL levels. It is of note that the gonadotropin LH exhibited a correlation with VO_2_max in team A, and with sprinting performance in team B. To our mind, the validity and physiological significance of this finding is questionable since in team A, LH correlated with VO_2_max performance in both experimental sessions but not with jumping and sprinting ability, whereas in team B, LH levels correlated with sprint performance (pre and post) but not with VO_2_max. In addition, despite the considerable changes in all performance parameters observed at the end of the detraining period, the LH levels did not exhibit any alterations. These discrepancies indicate that these data are of minor physiological significance, and do not necessarily constitute a significant correlation between LH and VO_2_max, 10 m, and 20 m performance.

## Conclusion

In conclusion, our findings demonstrate that the six-week detraining period, in our study, resulted in significant declines in aerobic, strength (as indicating by SJ and CMJ), and sprint performance adaptations. Furthermore, it had a negative effect on body composition as indicated by the observed increases in body weight and body fat percentage at the end of the study. The off-season transition period did not result in any significant differences in the measured sex steroid concentrations. Moreover, apart from the observed relationship of minor physiological significance between LH with VO_2_max and sprinting capacity, no other correlations were evident between the circulating sex steroids levels and the values of the performance parameters. Therefore, our findings suggest that sex steroids were non-contributing parameters for the reductions in exercise performance indices, and the negatively altered body composition. The implications of our findings strongly suggest that professional soccer players should devise transition off-season periodization training programs that provide adequate mental and physical recovery after the demanding competitive season, but also allow these athletes to maintain aerobic, strength, and sprint adaptations, and retain optimal body composition status.
